# An Unusual Mechanism Of Sustained Right Atrial Tachycardia

**Published:** 2011-11-15

**Authors:** Krishna Kumar Mohanan Nair, Narayanan Namboodiri, Anees Thajudeen, Ajitkumar Valaparambil, Jaganmohan A Tharakan

**Affiliations:** Department of Cardiology, Sree Chitra Tirunal Institute for Medical Sciences and Technology, Trivandrum, Kerala, India

**Keywords:** right atrial tachycardia, tachycardia mechanism

## Abstract

Lower loop re-entry (LLR) flutter is a rare type of atypical right atrial flutter. Most of the reported cases occurred in association with typical flutter patterns as a transient arrhythmia. Our case is unique in the fact the LLR was sustained and persisted independently.

## Case report

**Figure 1:**
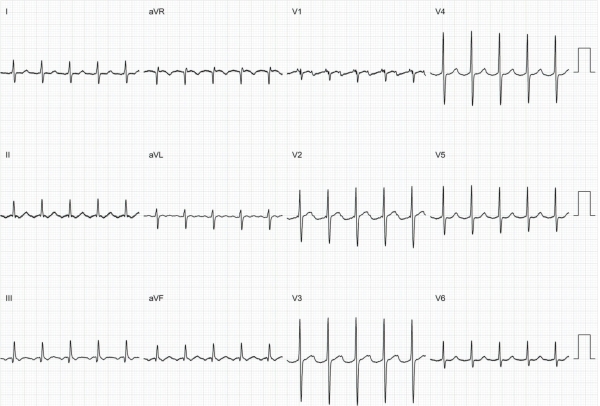
During tachycardia (ventricular response at 125/min), the P wave morphology was negative in inferior leads and positive-negative in V1

A 54-year-old male presented with a history of palpitation in the preceding 3 years, not controlled by oral metoprolol. During tachycardia (ventricular rate 125/min), the P wave morphology was negative in inferior leads and positive-negative in lead V1 (Figure 1).

**Figure 2:**
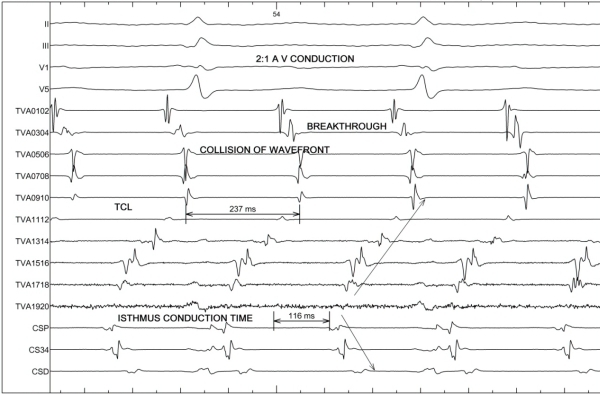
LLR With 2:1 AV conduction. Collision of wavefronts at upper lateral RA (TVA 5, 6) and break through at lower lateral RA (TVA 3, 4) suggestive of transcristal conduction

**Figure 3:**
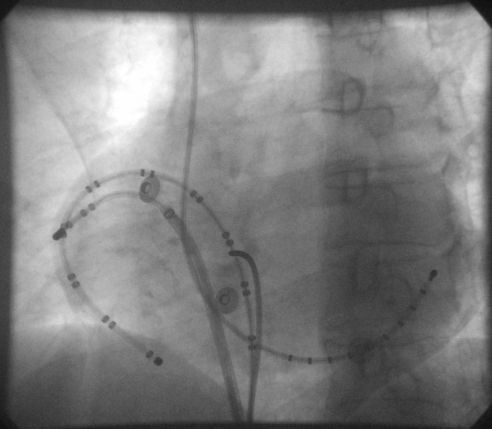
Fluoroscopy in LAO view showing TVA 1-2 lateral to CTI, TVA 19-20 medial to CTI and decapolar catheter in coronary sinus

At the time of the invasive electrophysiological study, the patient was having a narrow QRS tachycardia with cycle length of 237ms and 2:1 A V conduction (Figure 2 and 3). The activation wavefront was propagating from proximal coronary sinus (CS) to distal CS; at the same time showing counterclockwise activation through the septum and upper lateral right atrium (RA). The lower lateral RA was activated earlier than upper lateral RA. The wave fronts were seen to collide in the high lateral RA while the tachycardia was supported by a circuit at the base of the RA. The activation pattern suggested the possibility of tachycardia wave front traversing the cavotricuspid isthmus (CTI) and posterior RA with early activation of lower lateral RA through transcristal conduction, and collision of the 2 resultant circumtricuspid wave fronts - counterclockwise and clockwise, along the septum and freewall in RA respectively - at the upper anterolateral RA region.

To confirm the proposed tachycardia mechanism, entrainment was performed from CTI, lower lateral RA, and posterior RA. This protocol showed concealed fusion with postpacing interval approximating tachycardia cycle length, suggesting a macroreentrant mechanism involving CTI, posterior RA and lower anterolateral RA. On pacing from the RA roof during tachycardia, at a pacing cycle length 20ms lower than that of tachycardia cycle length, neither concealed fusion nor similar post pacing interval could be demonstrated, excluding RA roof a part of this re-entrant circuit. Hence a lower loop re-entry involving CTI, lower lateral RA and low posterior RA, mediated by the breaks in conduction at  lower cristae terminalis was considered.

Unlike to its original description where LLR intermittently converted to typical counterclockwise flutter, the reentry in this case was sustained and did not convert to typical flutter at any point during the study. Moreover, no other forms of CTI dependent flutters were inducible. The flutter terminated with linear ablation of CTI, and bidirectional block across the CTI was achieved with a few more burns. An aggressive pacing protocol after ablation failed to reinduce the tachycardia.

## Discussion

LLR is a rare type of RA flutter involving Tricuspid annulus-Eustachian ridge isthmus, lower lateral RA and posterior RA, and is maintained by circus movement of the activation wave front around the inferior vena cava instead of around the tricuspid annulus as in typical right atrial flutter [1,2]. Proof that these circuits were isthmus dependent was shown by concealed entrainment and/or response to CTI ablation. Usually it occurred spontaneously from pre-existing typical flutter patterns due to the intermittent transcristal conduction [1]. The transcristal conduction responsible for the early breakthrough in LLR is due to lack of persistent conduction barrier at the distal crista terminalis [1]. Due to the functional delay or block occurring at the distal crista, which may be intermittently hampering the transcristal conduction, LLR may convert to typical flutter intermittently. The fact that LLR persisted independently in this case, may be attributed to the existence of persistent transcristal conduction at the distal crista and to a linking phenomenon, in which the upper portions of the RA are excluded from the circuit by repetitive collision.

Yang et al [2] described 24 cases of LLR flutter in which 20 episodes were sustained. In 13 of the 24 episodes, the early breakthrough occurred at lower lateral RA, as in our case. They found that more than one annular break could occur at the lateral or anterolateral regions of the annulus. Yang et al [3], in another series, noted spontaneous transitions from atypical AFL to atrial fibrillation (AF). In the later series, a pattern of lower loop re-entry was observed prior to AF in 11 of 13 patients. Unlike the cases described in these series, the LLR in this report was sustained and did not convert to typical flutter.

However, the possibility of a potential typical flutter circuit involving CTI and peritricuspid region, apart from the LLR circuit, cannot be categorically excluded in this case.  As we ablated the CTI, the common isthmus of both re-entrant circuits, this was anyway non-inducible after the termination of LLR.  It is also likely that we could observe only LLR during the time of electrophysiological study, though other CTI-dependent flutter mechanisms could have been existent at different points in time.

## References

[R1] Cheng J (1999). Right atrial flutter due to lower loop reentry: mechanism and anatomic substrates. Circulation.

[R2] Yang YF (2001). Atypical right atrial flutter patterns. Circulation.

[R3] Yang Y (2003). Mechanism of conversion of atypical right atrial flutter to atrial fibrillation. Am J Cardiol.

